# Using affective judgement to increase physical activity in British adults

**DOI:** 10.1093/heapro/dax004

**Published:** 2017-02-27

**Authors:** Alice S Forster, Penny Buykx, Neil Martin, Susannah Sadler, Ben Southgate, Lauren Rockliffe, Ian Walker

**Affiliations:** 1 Research Department of Behavioural Science and Health, UCL, Gower Street, London, UK; 2 School of Health and Related Research, University of Sheffield, Regent Court, 30 Regent Street, Sheffield, UK; 3 Balance North East, Bede House, Ground Floor East, Unit 3, Belmont Business Park, Durham, UK; 4 HIGHBEAM LTD, Station Road, Cambridge, UK; 5 Department of Psychology, University of Bath, Bath, UK

**Keywords:** motor activity, exercise, mobile applications, affect, habits

## Abstract

Mobile phone apps have been shown to increase physical activity (PA), but existing apps fail to target the emotional aspects of PA, which influence whether individuals are active. We developed an app that encourages individuals to focus on the emotional aspects of PA. We aimed to assess the acceptability of this app, and conduct a preliminary evaluation of efficacy. The app was developed in collaboration with users through focus groups. Seven users tested the app over 4 months and provided feedback on acceptability, aesthetics and functionality in a follow-up focus group. Results were summarized descriptively. Before testing the app, participants completed a questionnaire assessing their current PA and psychological antecedents of PA. A second questionnaire was completed at the follow-up focus group. Change scores are reported for each participant and overall.

The social and reminder aspects facilitated motivation to be active and many found it easy to integrate into their lives. Most suggested modifications. Small improvements in number of minutes spent walking per week were observed (overall mean change +25 min) and some psychological antecedents of PA (overall mean change for social support for PA +0.14, self-efficacy for PA +0.17, outcome expectations about PA +0.20; all five-point scales), but reductions were seen in other domains. The app was acceptable to users, although developments are required. Testing with a small number of individuals, offering preliminary evidence of efficacy of this app, provides justification for further evaluation on a larger scale.

## INTRODUCTION

Evidence overwhelmingly suggests that most adults are not meeting physical activity (PA) recommendations (around 33% of men and 46% of women, Health Survey for England, 2013). Individuals who lead inactive lifestyles are at increased risk of developing some cancers (notably breast, bowel and womb cancers; IARC, 2002; WCRF, 2007; Wolin *et al.*, 2009; Moore *et al.*, 2010; Wu *et al.*, 2013). PA also helps individuals maintain a healthy body weight, and it is known that being overweight or obese increases risk of developing a number of diseases (Bianchini *et al.*, 2002; IARC, 2002; Reeves *et al.*, 2007; Renehan *et al.*, 2008; Blair, 2009; Hu, 2011). People who are active are more likely to live longer than those who are inactive (Andersen *et al.*, 2000; Wolin *et al.*, 2009; Samitz *et al.*, 2011). In 2012, it was estimated that 9% of all deaths worldwide were attributable to physical inactivity (Lee *et al.*, 2012).

Key barriers to the uptake of any new behaviour, particularly one like a PA regime requiring effort, are remembering, finding time and being motivated to do it. A promising long-term solution is to make the behaviour habitual. Habit theory [see (Walker *et al.*, 2014)] says that through repetition in a stable context, behaviours, which were initially the result of purposeful choice, can become automated or ‘scripted’ (Fujii, 2003), such that the behaviour is eventually initiated almost reflexively by external cues (cf. the unhealthy habit of a person who unthinkingly looks in the fridge every time they enter the kitchen). Habitual behaviours are likely to emerge when triggered by circumstances, rather than when a person feels sufficiently motivated, and so potentially reduces the need for motivation to be present. Habit theory also sees a parallel role for identity, which might equally be relevant here: seeing oneself as ‘the kind of person who is active’ should operate as a second spur to maintain activity. A central finding of habit research is that repetition is critical for habits to form (Lally *et al.*, 2010), and so interventions that support the regular repetition of activity during the first few weeks (Lally *et al.*, 2010; Walker *et al.*, 2014) before the behaviour is automated are suggested/recommended. Recent evidence suggests a distinction between exercise instigation habit and exercise execution habit, with instigation habit being strongly predictive of future exercise frequency (Phillips and Gardner, 2016). Change in exercise frequency has been found to be associated with exercise instigation, so it is plausible that interventions that aim to form exercise instigation habits may increase exercise frequency.

Given this promising role for habit to maintain PA, we then have the issue of how to initiate new activity in the first place. Affective judgement is defined as judgements about ‘the overall pleasure/displeasure, enjoyment, and feeling states expected from enacting an activity’ [(Nasuti and Rhodes, 2013), p. 358]. The likelihood of engaging in PA and self-reported affective states during PA is predicted by prior expectations of affect, with low expectations associated with low engagement and low pleasure (Ekkekakis, 2008; Rhodes *et al.*, 2009) and self-determination theory (SDT) also asserts that expectations about these states may influence future behaviour (Rhodes *et al.*, 2009; Ryan *et al.*, 2009). Modifying affective judgements, according to the cognitive evaluation theory, a sub-theory of SDT, may thereby potentially alter intrinsic motivation and ultimately PA.

PA mobile phone applications (apps) have shown promise in increasing PA (Foster *et al.*, 2013; Richards *et al.*, 2013), but existing apps largely target individuals who gain pleasure from quantifying their PA. They often have a competitive element (miles run, steps walked) and are usually tied to sport, rather than activity in general. Arguably, current apps ignore the pleasure that one might experience from the activity itself.

To fill this gap, we developed a novel PA app that, informed by the theoretical frameworks described above, aims to change PA behaviour in sedentary adults by modifying affective judgements about PA (to initiate new behaviour patterns) and prompting habit formation (to maintain these). The app is designed to reinforce the link between PA and positive mood by making salient positive affective judgements during and after activity. It does this by encouraging users to record and share positive images and feelings related to PA at around the time of that activity. We hypothesized that linking positive affect with activity in this way would increase the frequency of, and habit strength for, PA in users. The app was co-designed by target users to increase the likelihood that the app was acceptable and adopted by users.

In this paper we describe the development of the app, report a process evaluation that assessed the acceptability of the app, and provide preliminary evidence of the efficacy of the app in increasing PA and improving its psychological antecedents.

## METHODS

**Fig. 1:**
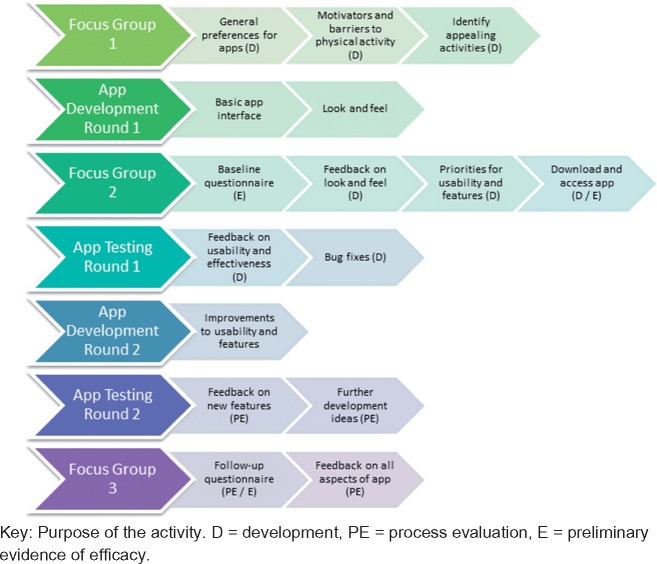
Process of developing the app, conducting the process evaluation and evaluating the app for preliminary evidence of efficacy.

The researchers developed the concept of the app and prepared an initial specification to develop the app. Subsequent development occurred in collaboration with users. The methods below describe the process of developing the app, conducting a process evaluation and evaluating the app for preliminary evidence of efficacy. These processes are denoted in Figure 1.

### Original specification

Our initial app specification described an app that reminds users to be physically active at a specific time and context each day (i.e. midday), coupled with a photograph that the user has previously posted denoting the positive feeling they experienced during a previous session of PA. Each time they are active, users would be reminded by the app to take a photograph that denoted the positive feeling they were experiencing and post it to the app. The specific PA behaviour was intended to be defined by the user during set-up of the app (although could be modified at a later date). Users would also be able to view the photos at any time. This was intended to remind them of their previously positive experience of PA to serve as additional motivation to get active should they need it. The app was designed to be used in the short to medium term, until a habit for a specific, user-defined PA has been formed.

### Developing the app

Co-design with potential users was a key principle of the app development process. The app was developed collaboratively, with users invited to attend a series of focus groups as well as to test the app. Potential users were recruited from an existing panel of participants held by a market research recruitment organization if they were aged 35–55, reported that their current PA level was sedentary, but were interested in changing this and owned a smartphone compatible with the prototype app (the app was initially developed for iOS), but were not using an existing PA app.

Three rounds of focus groups were conducted. In round one, users were asked to discuss their app needs and wants (apps in general and PA apps), their preferences for functionality, what limits their PA and what activities they felt were feasible for them. Two groups (16 users in total) were recruited for the first round to allow for attrition at subsequent rounds. At the second user group, users downloaded the app to their phones and were asked to discuss its acceptability, appropriateness, aesthetics and functionality. Nine users attended one group for the second round (all had attended one of the first round meetings). Most attendees at the second round group were female (78%) and the mean age was 47. User groups were facilitated by two members of the project team.

The app was built by a specialist, experienced app designer and coder, under the guidance of the project team. The app was developed iteratively, with user feedback integrated at each iteration (there were four rounds of development). Qualitative data collected while developing the app were not formally analysed, but summarized and key features integrated into the app design.

### Process evaluation

We collected process measures with regard to the use and acceptability of the final app. In between the second and third rounds of user groups, users were asked to test the app in their own time. Users reported feedback to the project team via email with any immediate comments they had about the app. They were also asked to discuss the acceptability, appropriateness, aesthetics and functionality of the app at the third round user group and to provide written feedback on frequency of use of the app, features that were liked/disliked, and beliefs about the app increasing PA in the individual and in others via a questionnaire. These process measures were summarized descriptively, with quotes provided where appropriate to give examples.

A key requirement of the app was that users could upload photographs that represented how being engaged in PA had made them feel. We collated these photographs and summarized the types of photographs that were uploaded. A coding frame was developed by LR and applied by LR and AF. Disagreements were resolved by discussion. Cohen’s *k* demonstrated that there was a good level of agreement between the two coders (*k* = 0.692, *p* < 0.0001).

### Preliminary testing of the efficacy of the app

Users attending the second user group were asked to complete a questionnaire at the start of the session. The questionnaire examined participants’ level of PA using four questions [adapted from Craig *et al.* (Craig *et al.*, 2003) and Health Survey for England (Health Survey for England, 2007)] and psychological outcomes that are related to PA: social norms [beliefs about what PA one’s friends and family do, three questions; based on Steadman *et al.* (Steadman *et al.*, 2002)], perceived social support to be physically active [three questions (Anderson *et al.*, 2006)]; self-efficacy/barriers (five questions, generated for this study), outcome expectations [five questions, modified from Anderson *et al.* (Anderson *et al.*, 2006)] and generated for this study; self-regulation [six questions, adapted from Anderson *et al.* (Anderson *et al.*, 2006)] and a measure of how strong a habit the participant has for being physically active [the 4 item Self-Report Behavioural Automaticity Index (Gardner *et al.*, 2012)]. Affective experience (when I am physically active I feel…), was assessed with a prompt for an open-ended response question created for this study, as existing measures did not meet our needs. At the third user group, users were asked to complete a final identical questionnaire (as noted above, users tested the app in between the second and third group, 4-month gap).

Mean scores were generated for constructs with multiple questions. Change scores for the measures of PA and psychological determinants of PA were calculated for each participant. We grouped participants by whether they posted to the app frequently based on the median number of photos posted (<9 low frequency user, ≥9 high frequency user). Mean change is also tabulated. Participants’ responses to the affective experience question were coded as positive or negative by LRand AF. All disagreements were resolved by discussion.

Ethical approval was obtained from the University College London Research Ethics Committee (6615001).

## RESULTS

### What the final app looks like

**Fig. 2:**
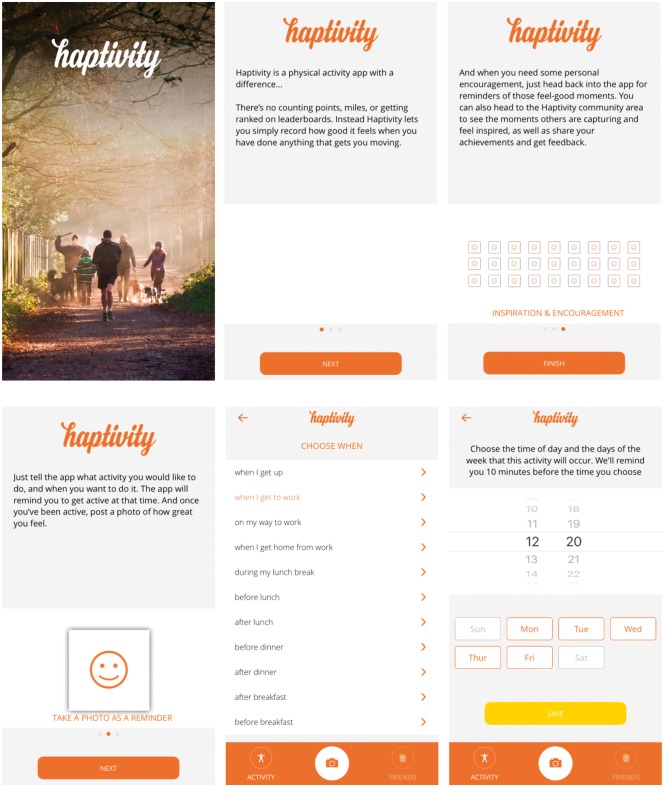
Images of the app and its functionality.

The app (‘Haptivity’; Figure 2). When users first open the app they were asked which specific PA behaviour they want to make a habit, when they would like to perform the behaviour and what they were likely to be doing at this time (i.e. before lunch). Users were encouraged to post a photograph that captures the positive emotions they experienced during PA (with a brief comment about why they felt good). Users were reminded to perform the behaviour at the time they had specified, and the reminder was accompanied by a previously posted photograph. Reminders could be turned on and off. Users’ posts could be seen publicly (future versions will allow users to control who can see their posts) and they could receive a restricted range of positive feedback provided by other app users. Feedback options were restricted to ensure that detrimental or negative comments were not posted. See Table 1 for behaviour change techniques linked to each component of the app. A broad range of behaviour change techniques were employed to maximise the likelihood of the app being efficacious. The social aspects of the app were not theoretically derived, but came from users’ preferences to share their photos and view/comment on others’ photos.

**Table 1:** Behaviour change techniques used in the app

App component	Behaviour change technique (Michie *et al.*, 2013)
Users reminded to be physically active at the same time and in the same context on a prescribed day/days	Habit formation
Users are encouraged to post photos that denote positive affect experienced during physical activity	Self-assessment of affective consequences
Users can view others’ posts	Social comparison
Users can provide a restricted range of positive feedback on others’ posts	Social reward

### Process evaluation

Seven users attended the third group and were generally positive about the app, but had suggestions for improvement and attitudes that suggested additional development would be required.

A key feature of the app is the sharing of PA photos with other users so that encouragement can be given, to facilitate future motivation. The encouragement element of the app did create PA motivation for some users, ‘It’s the same as if you were running a race. If people who are supporting you along the way, if you think to yourself, “I’ll give up,” but then somebody’s going to you, “Come on! No, you can do it!” it pushes you’. The ‘activity reminder’ function of the app was also well received and appeared to be successful in encouraging activity, ‘The reminders reminded me to do something… I actually felt like, because it’s come up I need to do it.’

Some users showed signs of ‘photo fatigue’, on the other hand. ‘I found the photos a bit mundane… including my own’. Contrarily, others thought that a text-based platform would be uninspiring ‘If it’s just the narrative, “I walked today,” no one’s gonna be looking at that’, suggesting that the app might need photos, but for these somehow to be more interesting, be that through promoting specific types of content or through enhancements added to the photos.

Users found the app simple to integrate into their lives. While some were particularly keen on the community/social motivation element of the app (‘When the app expired I kind of missed it and relieved when I was back on… I found myself looking to see who have given me motivation’), others questioned the added value in relation to current social media that they engage with (‘I’ll go on Facebook every day … and if I put in pictures of something I’ve done it’s usually on Facebook… I’d have to remember to go onto this app’). This suggests that further development of the app needs to ensure that the app offers something different from existing social media platforms.

The ability to quantify the PA performed, whilst not essential for some, would enhance the app’s value for others, ‘Counting calories, counting your steps… I suppose you want to know your progress and your end result’. Notably, these requests for numbers-based functionality was in opposition to the original aim of the app (to target affective judgement), and again suggests that users were demanding ‘something else’ to motivate them to use the app.

### Photographs uploaded to the app

The photos uploaded to the app fell into six main categories: people, PA, the outdoor environment, the indoor environment, lifestyle and other. These higher-order themes, along with lower level codes and total numbers of pictures uploaded, are presented in Supplementary Material.

Most photos focused on human subjects, with the majority capturing headshots or portraits of the users. Photos of the outdoors were also commonly uploaded, as were photos depicting PA and fitness. These included photos of the user or their friends and family engaging in some form of PA, as well as images of fitness equipment such as trainers, gym bags and workout clothes.

### Preliminary testing of the efficacy of the app

Seven participants completed a questionnaire at baseline and follow-up (Table 2; Supplementary Material). Three were low frequency users and four high frequency users.

**Table 2:** Change from baseline to post-app use

	Participant number									
	Low frequency users				High frequency users					
	1	2	3	Mean	4	5	6	7	Mean	Overall mean
Change in general physical activity (4-point scale^a^)	0.00	−1.00	Missing	−0.50	Missing	0.00	0.00	1.00	0.33	0.00
Change in vigorous activity (minutes per week)	40.00	690.00	0.00	243.33	50.00	−20.00	−90.00	−720.00	−195.00	−7.14
Change in moderate activity (minutes per week)	−15.00	Missing	0.00	−7.50	−140.00	15.00	−75.00	−30.00	−57.50	−40.83
Change in walking (minutes per week)	0.00	20.00	275.00	98.33	−135.00	40.00	−25.00	0.00	−30.00	25.00
Change in habit strength (range 1–5^a^)	−1.00	0.00	0.00	−1.00	−1.75	1.00	Missing	−0.50	−0.83	−0.38
Change in social support (range 1–5^a^)	0.67	0.00	1.33	0.67	0.33	0.00	−0.67	−0.67	−0.25	0.14
Change in descriptive norms (range 1–5^a^)	−1.00	0.67	0.00	−0.11	−0.67	0.33	0.00	0.00	−0.08	−0.10
Change in self efficacy (range 1–5^a^)	0.00	0.00	0.20	0.07	1.20	−0.80	−0.20	0.80	0.25	0.17
Change in outcome expectation (range 1–5^a^)	0.20	0.40	−0.20	0.13	0.00	−0.20	0.00	1.20	0.25	0.20

a Original question response option.

### PA outcomes

High frequency users reported being slightly more physically active overall at follow-up compared with baseline and low frequency users slightly less so (low frequency mean change: −0.50 on 4-point scale; high frequency: +0.33). Minutes of vigorous PA per week increased in the low frequency users (mean change: +243.33 min) but decreased in the high frequency users (−195.00) and overall (−7.14). Both low and high frequency users reported decreasing the number of minutes per week they spent doing moderate PA (low frequency mean change: −7.50, high frequency: −57.50). The groups went in opposite directions with the change in the number of minutes per week they spent walking (low frequency mean change: +98.33; high frequency: −30.00; overall: +25.00).

### Psychological outcomes

For low frequency users there was a small mean decrease (−0.10 on a 5-point scale) in the extent to which PA was a strong habit for them from before using the app to afterwards and as well as for high frequency users (−0.83, with small mean decrease across all participants of −0.38). Low frequency users reported slightly more social support to be physically active at follow-up (mean change +0.67), but high frequency users reported less social support (high frequency users: −0.25). Overall users reported slightly greater social support (+0.14). All users were less likely to perceive that others were physically active at follow-up compared with before they used the app (low frequency users mean change: −0.11, high frequency users mean change −0.08; overall change: −0.10). Low frequency users were more likely to perceive that they had self-efficacy to be physically active and had more positive outcome expectations about being physically active at follow-up compared with before they had used the app (mean change: self-efficacy: +0.07; outcome expectations: +0.13). High frequency users also had slightly more positive outcome expectations at follow-up (+0.25), and higher self-efficacy to be active (+0.25).

At baseline, when responding to the affective experience question, three participants reported a positive emotion associated with PA (one reported a positive and negative emotion, three did not respond) compared with four at follow-up (two reported a positive and negative emotion and one reported a negative emotion).

## DISCUSSION

In this paper we described the development of a behaviour change app that aims to facilitate the formation of a habit for PA by modifying affective judgement. The app was acceptable to users and qualitative results suggest that it motivated them to be active. The process evaluation demonstrated that further modification is required to enhance users’ motivation for using the app, so that it offers more that existing social media apps (which are often already integrated into people’s lives). Results provide preliminary evidence of the effect of the app on the psychological antecedents of PA as well as PA itself. Small improvements were seen in some domains, including minutes spent walking per week, but reductions in other areas (particularly amount of moderate and vigorous PA performed per week).

As a preliminary evaluation of efficacy, this study was not powered to detect change in PA or the psychological antecedents of being physically active. Nevertheless, results suggested some small improvements, as well as reductions, in the domains studied. There was large variability between individuals, some of which may be explained by how frequently participants used the app (defined as frequency of uploading a photo to the app). It is positive that improvements were observed for amount of walking performed per week, as this is likely to be an achievable activity for a previously sedentary population. Our preliminary data adds to the existing limited number of studies demonstrating that environmental and experiential PA interventions can have an effect on affective judgement (Rhodes *et al.*, 2009). However, future evaluation is warranted, employing a larger sample and testing at different times of the year (testing was conducted in winter in the UK and PA may be easier in warmer weather). It will be important that future testing considers the utility/acceptability of each of the app functions, rather than just the efficacy of the app as a whole, to ensure that each component is acceptable and working in the way that theory would predict. Future evaluation must also examine whether habits are maintained when the app is no longer used. It is possible that the reminder function will act as a cue for the behaviour, which when withdrawn may affect behaviour maintenance. However, the reminders appear at a user-specified time and context (e.g. before lunch), which will remain stable if the app is not used.

A key feature of the app is that it has to meet the needs of users as well as the aims of the project (users need to be motivated to use the app, so that the behaviour change techniques within the app can be employed), so additional components were added to the original specification. Such components, although not theoretically derived, were behaviour change techniques in themselves and may work to enhance overall efficacy. The social media function of the app is in competition with existing apps (such as Facebook and Instagram). Intuitively it seems that it would be foolish to compete with such well financed apps and future development of the app needs to ensure that it offers users something different from existing apps.

The study used a small sample of engaged individuals, which may limit the generalizability of the findings. The same group of individuals were used for both focus groups and the preliminary testing, meaning that participants were aware of the study objective when testing the app and their responses may have been subject to social desirability bias. The results may be an underestimation of the effect of the app. The app was developed for one platform, but will be expanded if appropriate following evaluation of efficacy. However its current form will have limited reach. Smartphone usage has increased, with over half of UK adults reporting ownership (Kantar World Panel, 2016). US adults from more deprived backgrounds are more likely to primarily access the internet from their phones compared with those from less deprived background (Gates *et al.*, 2014), suggesting that the app should not increase health inequalities. The app was developed on a small budget (£20 000 for research costs and no salary costs). As a result, we were unable to make all changes requested by the users or match the functionality of other apps the users used. The preliminary efficacy data and psychosocial determinants of exercise were not collected using objective measures. Others have also raised concerns about measuring habit using self-report (Hagger *et al.*, 2015).

## CONCLUSIONS

This behaviour change app, that attempts to modify affective judgement to form a habit for PA was acceptable to users. There was preliminary evidence that the intervention increased the amount that users walked per week, although reductions in other domains were observed. Small improvements (as well as reductions) were also seen for the psychological antecedents of PA. Additional development is required, followed by further evaluation of efficacy with a larger sample.

## Supplementary Material

Supplementary Data

## References

[dax004-B1] Andersen L. B., Schnohr P., Schroll M., Hein H. O. (2000) All-cause mortality associated with physical activity during leisure time, work, sports, and cycling to work. Archives of Internal Medicine, 160, 1621–1628. 10847255 10.1001/archinte.160.11.1621

[dax004-B2] Anderson E. S., Wojcik J. R., Winett R. A. and Williams D. M. (2006) Social-cognitive determinants of physical activity: the influence of social support, self-efficacy, outcome expectations, and self-regulation among participants in a church-based health promotion study. Health Psychology, 25, 510–520. 16846326 10.1037/0278-6133.25.4.510

[dax004-B3] Bianchini F., Kaaks R., Vainio H. (2002) Overweight, obesity, and cancer risk. Lancet Oncology, 3, 565–574. 12217794 10.1016/s1470-2045(02)00849-5

[dax004-B4] Blair S. N. (2009) Physical inactivity: the biggest public health problem of the 21st century. British Journal of Sports Medicine, 43, 1–2. 19136507

[dax004-B5] Craig C. L., Marshall A. L., Sjostrom M., Bauman A. E., Booth M. L., Ainsworth B. E. et al (2003) International physical activity questionnaire: 12-country reliability and validity. Medicine & Science in Sports & Exercise, 35, 1381–1395. 12900694 10.1249/01.MSS.0000078924.61453.FB

[dax004-B6] Ekkekakis P. (2008) Affect circumplex redux: the discussion on its utility as a measurement framework in exercise psychology continues. International Review of Sport and Exercise Psychology, 1, 139–159.

[dax004-B7] Foster C., Richards J., Thorogood M., Hillsdon M. (2013) Remote and web 2.0 interventions for promoting physical activity. Cochrane Database of Systematic Reviews, 9, CD010395. 24085594 10.1002/14651858.CD010395.pub2 PMC9674455

[dax004-B8] Fujii S. G. T. (2003) Development of script-based travel-mode choice after forced change. Transportation Research Part F: Traffic Psychology and Behaviour, 6, 117–124.

[dax004-B9] Gardner B., Abraham C., Lally P., de Bruijn G. J. (2012) Towards parsimony in habit measurement: testing the convergent and predictive validity of an automaticity subscale of the Self-Report Habit Index. International Journal of Behavioral Nutrition and Physical Activity, 9, 102. 10.1186/1479-5868-9-102 PMC3552971 22935297

[dax004-B10] Gates A., Stephens J. and Artiga S. (2014). Profiles of Medicaid Outreach and Enrollment Strategies: Using Text Messaging to Reach and Enroll Uninsured Individuals into Medicaid and CHIP. http://kff.org/medicaid/issue-brief/profiles-of-medicaid-outreach-and-enrollment-strategies-using-text-messaging-to-reach-and-enroll-uninsured-individuals-into-medicaid-and-chip/#endnote_link_101364-6 (last accessed 7 April 2016).

[dax004-B11] Hagger M. S., Rebar A. L., Mullan B., Lipp O. V., Chatzisarantis N. L. (2015) The subjective experience of habit captured by self-report indexes may lead to inaccuracies in the measurement of habitual action. Health Psychology Review, 9, 296–302. 25189762 10.1080/17437199.2014.959728

[dax004-B12] Health Survey for England. (2007) Healthy lifestyles: knowledge, attitudes and behaviour. http://www.hscic.gov.uk/catalogue/PUB00415/heal-surv-life-know-atti-beha-eng-2007-rep-v2.pdf (last accessed 7 April 2016).

[dax004-B13] Health Survey for England. (2013) Health Survey for England - 2013, Trend tables: Adult trend tables. https://www.gov.uk/government/statistics/health-survey-for-england-trend-tables-2013 (last accessed 16 February 2015).

[dax004-B14] Hu F. B. (2011) Globalization of diabetes: the role of diet, lifestyle, and genes. Diabetes Care, 34, 1249–1257. 21617109 10.2337/dc11-0442 PMC3114340

[dax004-B15] IARC. (2002) Weight control and physical activity In Vainio H., Bianchini F. (eds), IARC Handbook of Cancer Prevention. IARC, Lyon, p. 6.

[dax004-B16] Kantar World Panel. (2016) Kantar World Panel. http://www.kantarworldpanel.com/global (last accessed 7 April 2016).

[dax004-B17] Lally P., van Jaarsfeld C. H. M., Potts H. W. W., Wardle J. (2010) How habits are formed: modelling habit formation in the real world. European Journal of Social Psychology, 40, 998–1009.

[dax004-B18] Lee I. M., Shiroma E. J., Lobelo F., Puska P., Blair S. N., Katzmarzyk P. T. (2012) Effect of physical inactivity on major non-communicable diseases worldwide: an analysis of burden of disease and life expectancy. Lancet, 380, 219–229. 22818936 10.1016/S0140-6736(12)61031-9 PMC3645500

[dax004-B19] Michie S., Richardson M., Johnston M., Abraham C., Francis J., Hardeman W., et al (2013) The behavior change technique taxonomy (v1) of 93 hierarchically clustered techniques: building an international consensus for the reporting of behavior change interventions. Annals of Behavioral Medicine, 46, 81–95. 23512568 10.1007/s12160-013-9486-6

[dax004-B20] Moore S. C., Gierach G. L., Schatzkin A., Matthews C. E. (2010) Physical activity, sedentary behaviours, and the prevention of endometrial cancer. British Journal of Cancer, 103, 933–938. 20877336 10.1038/sj.bjc.6605902 PMC2965881

[dax004-B21] Nasuti G., Rhodes R. E. (2013) Affective judgment and physical activity in youth: review and meta-analyses. Annals of Behavioral Medicine, 45, 357–376. 23297073 10.1007/s12160-012-9462-6

[dax004-B22] Phillips L. A., Gardner B. (2016) Habitual exercise instigation (vs. execution) predicts healthy adults' exercise frequency. Health Psychology, 35, 69–77. 26148187 10.1037/hea0000249

[dax004-B23] Reeves G. K., Pirie K., Beral V., Green J., Spencer E., Bull D. (2007) Cancer incidence and mortality in relation to body mass index in the Million Women Study: cohort study. British Medical Journal, 335, 1134. 17986716 10.1136/bmj.39367.495995.AE PMC2099519

[dax004-B24] Renehan A. G., Tyson M., Egger M., Heller R. F., Zwahlen M. (2008) Body-mass index and incidence of cancer: a systematic review and meta-analysis of prospective observational studies. Lancet, 371, 569–578. 18280327 10.1016/S0140-6736(08)60269-X

[dax004-B25] Rhodes R. E., Fiala B. and Conner M. (2009) A review and meta-analysis of affective judgments and physical activity in adult populations. Annals of Behavioral Medicine, 38, 180–204. 20082164 10.1007/s12160-009-9147-y

[dax004-B26] Richards J., Hillsdon M., Thorogood M., Foster C. (2013) Face-to-face interventions for promoting physical activity. Cochrane Database of Systematic Reviews, 9, CD010392. 10.1002/14651858.CD010392.pub2 PMC11542891 24085592

[dax004-B27] Ryan R. M., Williams G. C., Patrick H., Deci E. L. (2009) Self-determination theory and physical activity: the dynamics of motivation in development and wellness. Hellenic Journal of Psychology, 6, 107–124.

[dax004-B28] Samitz G., Egger M. and Zwahlen M. (2011) Domains of physical activity and all-cause mortality: systematic review and dose-response meta-analysis of cohort studies. International Journal of Epidemiology, 40, 1382–1400. 22039197 10.1093/ije/dyr112

[dax004-B29] Steadman L., Rutter D. R. and Field S. (2002) Individually elicited versus modal normative beliefs in predicting attendance at breast screening: examining the role of belief salience in the theory of planned behaviour. British Journal of Health Psychology, 7(Part 3), 317–330. 12614503 10.1348/135910702760213706

[dax004-B30] Walker I., Thomas G. O., Verplanken B. (2014) Old habits die hard: travel habit formation and decay during an office relocation. Environment and Behaviour, 47, 1089–1106.

[dax004-B31] WCRFA. (2007) Food, Nutrition, Physical Activity and the Prevention of Cancer: A Global Perspective. American Institute for Cancer Research, Washington.

[dax004-B32] Wolin K. Y., Yan Y., Colditz G. A., Lee I. M. (2009) Physical activity and colon cancer prevention: a meta-analysis. British Journal of Cancer, 100, 611–616. 19209175 10.1038/sj.bjc.6604917 PMC2653744

[dax004-B33] Wu Y., Zhang D. and Kang S. (2013) Physical activity and risk of breast cancer: a meta-analysis of prospective studies. Breast Cancer Research and Treatment, 137, 869–882. 23274845 10.1007/s10549-012-2396-7

