# Supplementary material for: Using affective judgement to increase physical activity in British adults
Source: Health Promot Int. 2017 Feb 27;33(4):648–56. doi: 10.1093/heapro/dax004 (PMC6144778; doi:10.1093/heapro/dax004)
Supplement: Supplementary Data [file dax004_supplementary_material.docx]

***Characteristics of the users attending the third focus group***

|  | **Participant number** | | | | | | |
| --- | --- | --- | --- | --- | --- | --- | --- |
|  | 1 | 2 | 3 | 4 | 5 | 6 | 7 |
| **Age** | 46 | 43 | 43 | 43 | 38 | 55 | 41 |
| **Gender** | Female | Female | Female | Female | Female | Male | Male |
| **Employment status** | Employed | Employed | Employed | Employed | Employed | Employed | Employed |
| **Number of photos posted to the app** | 3 | 4 | 7 | 22 | 9 | 21 | 10 |
| **Values recorded at the 2^nd^ focus group** |  |  |  |  |  |  |  |
| General physical activity  (4 point scale) | 3 | 2 | Missing | 3 | 3 | 3 | 3 |
| Vigorous activity (minutes per week) | 0 | 10 | 0 | 10 | 40 | 120 | 240 |
| Moderate activity (minutes per week) | 15 | Missing | 0 | 30 | 15 | 120 | 30 |
| Walking  (minutes per week) | 90 | 10 | 5 | 45 | 20 | 15 | 30 |
| Habit  (Range 1-5) | 2.50 | 1.67 | 1.00 | 3.50 | 1.00 | Missing | 3.17 |
| Social support  (Range 1-5) | 3.00 | 1.67 | 3.00 | 1.67 | 2.33 | 2.00 | 2.67 |
| Descriptive norms  (Range 1-5) | 2.67 | 5.00 | 3.00 | 2.67 | 2.67 | 2.00 | 4.33 |
| Self-efficacy  (Range 1-5) | 2.40 | 3.40 | 2.80 | 2.20 | 3.00 | 2.80 | 3.00 |
| Outcome expectation  (Range 1-5) | 1.40 | 2.20 | 1.80 | 1.80 | 1.80 | 1.20 | 3.80 |

***Higher order themes, codes and number of photos uploaded.***

| **Higher order themes** | **Codes** | **Number of photos** |
| --- | --- | --- |
| People | Headshot | 16 |
|  | Portrait | 5 |
|  | Body part | 2 |
|  | Group gathering | 1 |
| Physical activity | Person/people engaging in PA | 4 |
|  | Workout equipment | 6 |
| The outdoor environment | Nature/landscape | 8 |
|  | Architecture | 7 |
|  | Night-time street view | 5 |
| The indoor environment | Home environment | 2 |
|  | Office environment | 1 |
| Lifestyle | Animal/pet | 2 |
|  | Vehicle | 1 |
|  | Food/meal | 4 |
| Other | Abstract image | 7 |
|  | Inanimate object | 3 |
|  | Uploaded/photographed image | 2 |
